# Pyruvate kinase M2 may be a therapeutic target in a mouse model of human non-small cell lung cancer

**DOI:** 10.1186/2051-1426-3-S2-P374

**Published:** 2015-11-04

**Authors:** Akiko Suzuki, Sachin Puri, Bharat H Joshi, Pamela Leland, Bernard A Fox, Raj K Puri

**Affiliations:** 1 Center for Biologics Evaluation & Research (CBER)/Food & Drug Administration (FDA), Silver Spring, MD, USA; 2 Molecular & Tumor Immunology/Robert W. Franz Cancer Research Center/Earle A. Chiles Research Institute/Providence Cancer Center, Portland, OR, USA; 3 Earle A. Chiles Research Institute, Portland, OR, USA

## 

Non-small cell lung cancer (NSCLC) is the most common cause of cancer related mortality worldwide and in need of new treatment options. Previously, we have shown that 97 genes are overexpressed in 9 primary NSCLC cell lines by DNA microarray technology. Pyruvate kinase M2 (PKM2), an allosteric isoform of pyruvate kinase and metabolic enzyme necessary for aerobic glycolysis and cell proliferation, was identified to be over-expressed in the NSCLC cell lines. Real-time qRT-PCR and immunohistochemistry (IHC) techniques confirmed the microarrays results. Consistent with the over-expression of PKM2, all 9 NSCLC cell lines showed higher PKM2 enzyme activity than normal lung cell lines. Since a chemical inhibitor of PKM2 decreased PKM2 enzyme activity and proliferation of NSCLC cell lines, we hypothesized that PKM2 inhibitors may have a role in antitumor effects *in vivo*. Therefore, we evaluated antitumor effects of a PKM2 inhibitor in murine models of NSCLC. Mice tolerated intra-tumoral administration of the PKM2 inhibitor (Small molecule inhibitor for PKM2) up to 500 µg/kg/day without any evidence of visible toxicity or weight loss. Despite its anti-proliferative activities *in vitro*, the PKM2 inhibitor did not mediate significant antitumor effects in NSCLC tumor xenografts. The mechanism of lack of activity *in vivo* is not clear. It is possible that PMK2 inhibitor levels were not sufficient or sustained *in vivo* for a measurable antitumor effect.

To further study whether PKM2 was a target for therapy, we silenced the PKM2 gene in the H1299 and H358 NSCLC cell lines using RNAi and proliferative activity of tumors was assessed *in vitro* and *in vivo*. Gene silencing in cells transfected with plasmids encoding shRNAs was confirmed by IHC, which showed >80% silencing measured by NIKON-S-Element software at the protein level. In clonogenic assays, we observed >50% fewer colonies in two PKM2 silenced NSCLC cell lines compared to mock transfected controls. In subcutaneous xenograft tumor models in athymic nude mice, preliminary results showed that PKM2-silenced tumors grew significantly slower compared to mock transfected tumors (p < 0.05). These results suggest that PKM2 may be a target for cancer therapy. Additionally, given its over-expression in NSCLC, it may also serve as a target for immunotherapy. Studies are ongoing to confirm these results, understand a mechanism of tumor response, and determine whether patients generate immune responses to PKM2.

